# On the road with WRAP53β: guardian of Cajal bodies and genome integrity

**DOI:** 10.3389/fgene.2015.00091

**Published:** 2015-03-24

**Authors:** Sofia Henriksson, Marianne Farnebo

**Affiliations:** ^1^Science for Life Laboratory, Division of Translational Medicine and Chemical Biology, Department of Medical Biochemistry and Biophysics, Karolinska InstitutetStockholm, Sweden; ^2^Department of Oncology-Pathology, Cancer Centrum Karolinska, Karolinska InstitutetStockholm, Sweden

**Keywords:** WRAP53, WDR79, TCAB1, Cajal body, telomerase, SMN, scaRNA, DNA repair

## Abstract

The *WRAP53* gene encodes both an antisense transcript (WRAP53α) that stabilizes the tumor suppressor p53 and a protein (WRAP53β) involved in maintenance of Cajal bodies, telomere elongation and DNA repair. WRAP53β is one of many proteins containing WD40 domains, known to mediate a variety of cellular processes. These proteins lack enzymatic activity, acting instead as platforms for the assembly of large complexes of proteins and RNAs thus facilitating their interactions. WRAP53β mediates site-specific interactions between Cajal body factors and DNA repair proteins. Moreover, dysfunction of this protein has been linked to premature aging, cancer and neurodegeneration. Here we summarize the current state of knowledge concerning the multifaceted roles of WRAP53β in intracellular trafficking, formation of the Cajal body, DNA repair and maintenance of genomic integrity and discuss potential crosstalk between these processes.

## Introduction

The eukaryotic cell nucleus is highly organized with several sub-compartments containing high concentrations of factors involved in specific biological processes to optimize performance. Numerous distinct, non-membrane-bound nuclear bodies, including nucleoli, nuclear speckles, histone locus bodies, promyelocytic leukemia (PML) bodies and Cajal bodies, have been identified and shown to overlap with respect to their components and organization. Formation of such sub-organelles usually involves dynamic processes, such as protein modifications and self-association, various RNA-protein interactions and tethering of central factors to specific gene loci (Dundr and Misteli, 2010; Machyna et al., 2013).

Identified more than a century ago and subjected to intense functional investigation, the Cajal body has been suggested to be associated with telomere maintenance and maturation of the splicing machinery (Machyna et al., 2013). In a similar manner, DNA repair factors are sequestered in specialized repair centers called foci. Following induction of DNA double-strand breaks, a variety of proteins are mobilized to the break sites to initiate the signaling cascades required for proper repair (Polo and Jackson, 2011).

WRAP53β (also denoted WRAP53 or WDR79 or TCAB1), is a scaffold protein that directs factors to Cajal bodies, telomeres and DNA double-strand breaks, thereby facilitating the interactions necessary for appropriate biological responses (Tycowski et al., 2009; Venteicher et al., 2009; Mahmoudi et al., 2010). The WD40 domain of WRAP53β appears to be critical for its function, by serving as a scaffold for multiple interactions between a wide variety of molecules. Mutations predicted to impair the structure of this domain cause dyskeratosis congenita, a syndrome associated with premature aging and an elevated predisposition for cancer, highlighting the importance of WRAP53β for homeostasis (Zhong et al., 2011).

In the present review, we focus on WRAP53β and its reported roles in the maintenance of the Cajal body, as a component of the telomerase enzyme and, recently, in DNA damage response and repair. We also discuss the involvement of this protein in various diseases.

## *WRAP53*: one gene—multiple products

**Figure 1:**
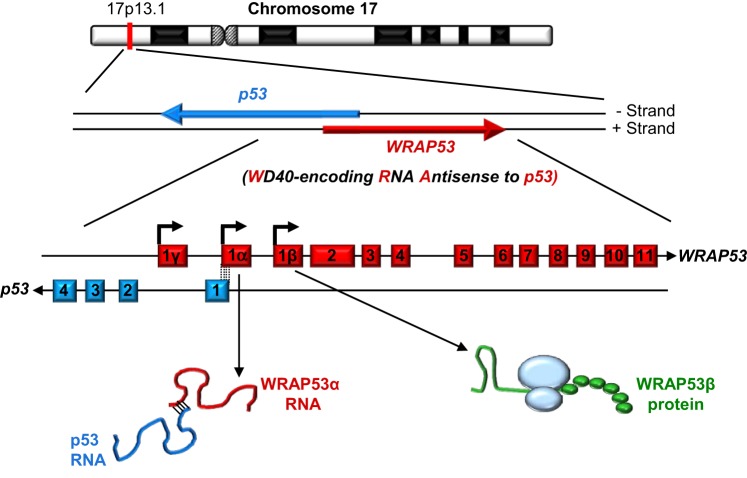
**The *WRAP53* and *p53* genes and their gene products**. The arrows indicate the direction of transcription and the dotted lines the exon overlap between these genes.

As its name indicates, the WD40-encoding RNA antisense to p53 (*WRAP53*) gene, identified in our laboratory, encodes at least two different functional products: an antisense transcript that stabilizes p53 (referred to as WRAP53α) and, via alternative transcriptional start site usage, a protein containing WD40 repeats (referred to as WRAP53β, alias WRAP53, WDR79 and TCAB1). Moreover, a third alternative start site in exon 1γ gives rise to WRAP53γ transcripts overlapping the first intron of p53, the function of which remains elusive and is not discussed further here (Figure 1) (Farnebo, 2009; Mahmoudi et al., 2009; Tycowski et al., 2009; Venteicher et al., 2009).

## WRAP53α: a natural p53 antisense transcript

The *WRAP53* gene on chromosome 17p13 partially overlaps the *p53* tumor suppressor gene in a head-to-head orientation (Figure 1). This organization has functional consequences, i.e., WRAP53α transcripts containing this overlap regulate the levels of p53 mRNA and protein.

By binding to the 5′ UTR region of p53 mRNA through a perfectly complementary sequence, WRAP53α stabilizes this mRNA, thereby promoting induction of the p53 protein in response to DNA damage required for mediating apoptosis (Mahmoudi et al., 2009). The insulator protein CTCF contributes to WRAP53α-mediated regulation of p53 by binding WRAP53α RNA (Saldana-Meyer et al., 2014). Several lines of evidence indicate that neither WRAP53β transcripts nor protein are involved in regulating p53: (1) overexpression of the overlapping exon 1α, which is not present in WRAP53β transcripts, efficiently elevates the steady-state level of p53: whereas overexpression of exon 1β or the WRAP53β protein has no such effect. (2) siRNAs targeting exon 1α, but not exon 1β down-regulate p53. And (3) knockdown of WRAP53α transcripts does not alter WRAP53β protein levels, but nevertheless reduces p53 expression (Farnebo, 2009; Mahmoudi et al., 2009).

## WRAP53β: an essential Cajal body component

**Figure 2:**
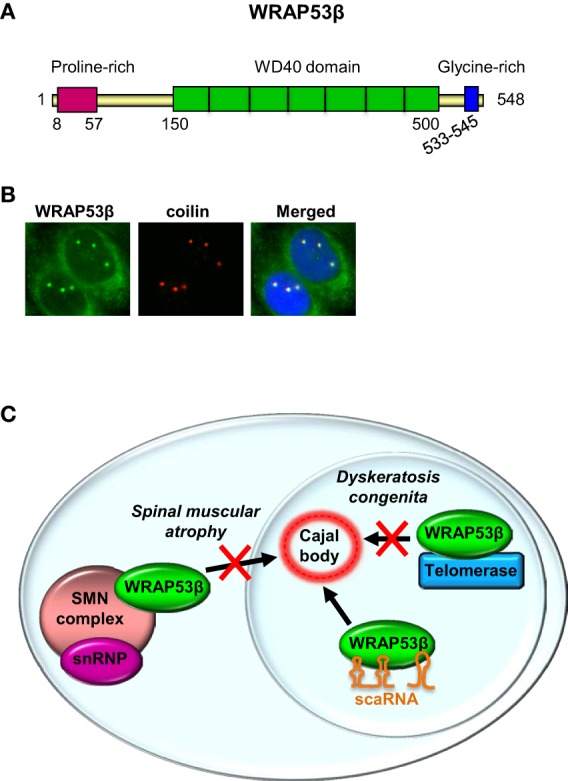
**WRAP53β is a WD40 protein and a hub in Cajal bodies. (A)** The structure of WRAP53β. The numbers indicate amino acid residues. **(B)** Immunostaining of WRAP53β and coilin, a marker for Cajal bodies, in U2OS cells (Mahmoudi et al., 2010). Nuclei were stained with DAPI. **(C)** Schematic illustration of WRAP53β-mediated trafficking of the SMN complex, scaRNAs and telomerase to Cajal bodies, and of diseases associated with loss of this function.

Most investigations on the *WRAP53* gene have focused on the WRAP53β (WRAP53/WDR79/TCAB1) protein, which is highly evolutionary conserved, with homologs (confined to its WD40 repeats) in vertebrates, invertebrates, plants and yeast. A WD40 repeat is a short structural motif containing approximately 40 amino acids and often a C-terminal tryptophan (W) and aspartic acid (D) dipeptide. Typically multiple WD40 repeats exist within a WD40 domain, which allow interaction with several partners simultaneously, in a non-exclusive manner (Stirnimann et al., 2010; Xu and Min, 2011). WRAP53β consist of a proline-rich N-terminus (16 of 50 residues = 32%), a central WD40 domain (predicted to contain 5–7 repeats) and a glycine-rich C-terminus (7 of 13 residues = 54%) (Figure 2A).

The WRAP53β protein is found both in the cytoplasm and highly enriched in nuclear organelles known as Cajal bodies (Figure 2B) (Mahmoudi et al., 2010). These spherical organelles (0.2–2 μM), were identified and described by Santiago Ramón y Cajal in 1903 (Gall, 2003). Their numbers range from 1 to 10 per nucleus, being highest in cells with rapid rates of transcription and splicing (Cioce and Lamond, 2005; Boulon et al., 2010).

Cajal bodies are characterized by the marker protein coilin that interacts with many factors and thereby serves as a platform for the assembly of Cajal bodies. Cajal bodies are rich in ribonucleoprotein (RNP) complexes, including the spliceosomal small nuclear RNPs (snRNPs), small Cajal body-specific RNPs (scaRNPs), small nucleolar RNPs (snoRNPs) and components of the telomerase RNP complex. Accordingly, Cajal bodies have been described to play essential roles in the maturation of snRNPs and snoRNPs and telomere maintenance. Furthermore, the survival of motor neuron (SMN) complex and factors involved in 3′-end processing of histone mRNA accumulate in these bodies (Carvalho et al., 1999; Machyna et al., 2013). Defects in Cajal body formation are linked to impaired cell proliferation and splicing, but rather than being essential for the associated processes, this compartment is thought to enhance efficiency by concentrating necessary factors in the same space (Lemm et al., 2006; Whittom et al., 2008; Novotny et al., 2011).

Cajal bodies are highly dynamic, moving within the nucleoplasm except when transiently immobilized through interaction with chromatin. These interactions occur at specific loci with repeated clusters of snRNA, U3 snoRNA and histone genes and in a transcription-dependent manner (Frey and Matera, 1995; Gao et al., 1997; Frey et al., 1999; Platani et al., 2000). Tethering experiments have revealed that immobilization of various constituents leads to the de novo formation of Cajal bodies, a self-organized process that appears to occur in random order, without internal hierarchy of individual components (Kaiser et al., 2008; Shevtsov and Dundr, 2011).

Several studies have emphasized the role of WRAP53β as a central player in maintenance of and localization of factors to the Cajal body (Figure 2C). Indeed, without WRAP53β these organelles collapse and cannot re-form. Exogenous WRAP53β accumulates in Cajal bodies, but does not stimulate *de novo* formation of this organelle. Instead, Cajal bodies are disrupted when high levels of this protein are exogenously expressed, indicating an adverse effect on the function of endogenous WRAP53β, potentially via self-association or sequestration of certain factors important for Cajal body formation (Mahmoudi et al., 2010). Similar effects have been observed when coilin is exogenously overexpressed (Hebert and Matera, 2000).

In addition to maintaining their structural integrity, WRAP53β targets several factors to Cajal bodies and loss of this trafficking is associated with various disorders (see further below). Depletion of this protein causes many factors to mislocalize to the nucleolus (Mahmoudi et al., 2010). This may indicate that WRAP53β promotes the translocation of factors from the nucleolus to Cajal bodies or alternatively by binding and directing factors to these bodies, prevents them from accumulating in nucleoli. WRAP53β does not regulate localization of factors to other nuclear organelles, such as gems, splicing speckles and PML bodies (Mahmoudi et al., 2010).

## WRAP53β guides SMN across the cell to Cajal bodies

One factor, which WRAP53β helps to localize to Cajal bodies, is the SMN protein that together with its partner proteins Gemin2-8 and Unrip, forms the SMN complex involved in the cytoplasmic assembly of the spliceosomal snRNPs (Gubitz et al., 2004; Chari et al., 2008; Cauchi, 2010; Matera and Wang, 2014). By binding Sm proteins and snRNAs separately, the SMN complex promotes Sm binding specifically to snRNA (Pellizzoni et al., 2002).

WRAP53β is involved in the translocation of SMN across the cell. After binding SMN in the cytoplasm, WRAP53β first recruits this protein to the nucleus by promoting its interaction with the nuclear pore receptor importinβ and then targets SMN to Cajal bodies by facilitating its interaction with coilin. Thus, knockdown of WRAP53β results in accumulation of SMN in the cytoplasm and mislocalization of nuclear SMN to the nucleolus. WRAP53β itself is targeted to Cajal bodies via its WD40 domain and a stretch of its C-terminus, and these same regions appears to scaffold the SMN-coilin interaction (Mahmoudi et al., 2010).

## Is the WRAP53β route blocked in spinal muscular atrophy?

Mutations in the *SMN1* gene results in spinal muscular atrophy (SMA), a neurodegenerative disorder characterized by progressive degeneration of spinal cord anterior horn α-motor neurons and the leading genetic cause of infant mortality with an incidence of approximately 1:6000 live births (Coady and Lorson, 2011). The reason why spinal motor neurons are particularly sensitive to mutations of the SMN protein is not fully understood. Cells lacking SMN display impaired assembly of the snRNP core, along with splicing defects (Fischer et al., 1997; Pellizzoni et al., 2002; Zhang et al., 2008), however, the fact that most human cells require splicing points to additional functions of SMN important for this cell type. In line with such an idea, the nuclear function of SMN remains unclear and the severity of the SMA disease increases as the level of SMN protein and number of SMN-containing nuclear structures decreases (Lefebvre et al., 1997; Oskoui et al., 2007; Tapia et al., 2012).

Interestingly, defective WRAP53β-mediated trafficking of SMN is observed in patients afflicted by the most severe form of spinal muscular atrophy (type I or Werdnig-Hoffmann disease) (Lefebvre et al., 1997; Oskoui et al., 2007; Mahmoudi et al., 2010; Tapia et al., 2012). This impaired interaction could not be explained by the lower amount of SMN protein present; instead, WRAP53β can apparently not bind properly to SMN and localize SMN to Cajal bodies in these cells (Mahmoudi et al., 2010). Since WRAP53β recently was identified as an important regulator of DNA double-strand break repair (Henriksson et al., 2014), it is possible that SMN collaborates with WRAP53β in this process and that impaired DNA repair contributes to the pathogenesis of SMA.

## Another highway: WRAP53β targets scaRNA to Cajal bodies

WRAP53β also guides a class of RNA molecules referred to as scaRNAs to Cajal bodies, which are required for catalyzing post-transcriptional modifications (including pseudouridylation and 2′-O-methylation) of the snRNA component of snRNPs (Tycowski et al., 2009). These modifications are important for their proper incorporation into the spliceosome (Darzacq et al., 2002; Jady et al., 2003).

The scaRNA family consists of at least 20 members, including the RNA component of telomerase (TERC). They are divided into two major classes: the C/D box scaRNAs, which contain the C (RUGAUGA) and D (CUGA) motifs and direct methylation of target snRNAs, and the H/ACA box scaRNAs, which contain the H (ANANNA) and ACA motifs and guide isomerization of uridine into pseudouridine. The C/D box scaRNAs associate with four core proteins: the methyltransferase fibrillarin, NOP56, NOP58 and 15.5K/NHPX, while the H/ACA box scaRNAs associate with four other core proteins: the pseudouridine synthase dyskerin, GAR1, NHP2, and NOP10. The RNA components of the scaRNPs direct the enzymes and associated proteins to their target RNAs via sequence complementarity (Kiss, 2002; Kiss et al., 2006).

The scaRNAs are targeted to Cajal bodies by a common element, referred to as the Cajal body localization signal or CAB box. The consensus sequence of this element is: ugAG for H/ACAs scaRNA and GU-rich dinucleotide repeats in the case of C/D box scaRNAs (Richard et al., 2003; Tycowski et al., 2009; Marnef et al., 2014). Although, the factor(s) responsible for this targeting long remained unknown, WRAP53β has now been shown to associate specifically with the CAB box of scaRNAs and promote their targeting to Cajal bodies. Thus, CAB box mutations that disturb binding to WRAP53β or depletion of this protein results in mislocalization of scaRNAs to nucleoli (Tycowski et al., 2009).

Although WRAP53β does not appear to bind snRNAs or snoRNAs that lack CAB box motifs it does bind to another class of RNAs, the AluACA RNAs (Jady et al., 2012). Interestingly, these RNAs originate from Alu repeats, are processed into RNA containing H/ACA and CAB boxes and also associate with dyskerin, NOP10, NHP2 and GAR1. Despite their CAB box motifs, the AluACA RNAs accumulate in the nucleoplasm rather than Cajal bodies and their function is not known.

## WRAP53β: the telomerase taxi

The telomerase RNP holoenzyme catalyzes the addition of telomeric repeats TTAGGG onto the ends of linear chromosomes. The minimal catalytic unit consists of a reverse transcriptase (TERT) and the TERC RNA template containing the sequence copied by TERT (Artandi and Depinho, 2010). WRAP53β associates with the TERC CAB box of the enzymatically active telomerase complex and promotes its localization to Cajal bodies (Venteicher et al., 2009). Since TERC is an H/ACA scaRNA, it also binds the scaRNP core proteins dyskerin, GAR1, NHP2 and NOP10 that play important roles in the stability, nuclear localization and proper assembly of telomerase RNP (Artandi and Depinho, 2010). Structural deviations and a lack of complementary target RNAs indicate that TERC is not directly involved in pseudouridylation of snRNAs (Mitchell et al., 1999; Trahan and Dragon, 2009; Egan and Collins, 2012).

The observation that WRAP53β binds all core components of telomerase (including TERC, TERT and dyskerin), but not to telomerase assembly factors (including NAF1, pontin and reptin), indicates that this protein is a component of the active telomerase enzyme. WRAP53β is required for telomerase localization to Cajal bodies, which associate with telomeres during S-phase (Jady et al., 2006; Tomlinson et al., 2006; Venteicher et al., 2009). Knockdown of WRAP53β disrupts targeting of TERC to both Cajal bodies and telomeres and, consequently, leads to progressive telomere shortening. Similarly, cells with TERC containing a CAB box mutation also display telomere shortening, probably due to mislocalization of TERC to the nucleolus (Venteicher et al., 2009; Egan and Collins, 2010). However, telomerase function and telomere elongation was recently shown to be unaffected in Cajal body-deficient coilin knockout cells. This suggest that Cajal bodies not are essential in this process, whereas certain Cajal body factors such as WRAP53β clearly play important roles in telomere homeostasis. Additional studies are required to clarify the previous suggested role for the Cajal body in telomere biology (Chen et al., 2015).

## WRAP53β and dyskeratosis congenita

Germline mutations in WRAP53β result in dyskeratosis congenita, a syndrome characterized by bone marrow failure, premature aging, predisposition for cancer and a triad of mucocutaneous features including oral leukoplakia, abnormal skin pigmentation and nail dystrophy (Zhong et al., 2011). This disease is caused by defective telomere maintenance, since approximately 50% of all cases carry mutations in core components of the telomerase enzyme or in telomere capping proteins, such as TERC, TERT, dyskerin, TIN2 and WRAP53β (Dokal, 2011; Zhong et al., 2011; Ballew and Savage, 2013).

Although patients with dyskeratosis congenita display very short telomeres, clinical characteristics, such as age at onset and disease severity are not strictly correlated to telomere length. Moreover, with certain associated TERT mutations telomerase activity is maintained (Vulliamy et al., 2011; Zaug et al., 2013). Therefore, additional perturbations, such as impaired stem cell function and defects in rRNA processing and DNA repair, might be involved in the etiology of dyskeratosis congenita (Ruggero et al., 2003; Mochizuki et al., 2004; Zhang et al., 2012b; Bellodi et al., 2013).

Mutations in WRAP53β are inherited in an autosomal recessive fashion and reside in highly conserved regions of its WD40 domain. These mutations reduce the nuclear level of WRAP53β, impair its trafficking of telomerase to telomeres, and subsequently lead to progressive shortening of telomeres in these patients (Zhong et al., 2011). Recently, the chaperonin CCT/TRiC was identified to be crucial for proper folding of WRAP53β and this folding was found to be impaired in dyskeratosis congenita (Freund et al., 2014). Since mutated and un-folded WRAP53β is not translocated into the nucleus, all of its activities in this organelle should be disturbed.

Interestingly, our group demonstrated recently that WRAP53β is involved in the repair of DNA double-strand breaks (Henriksson et al., 2014), which thus might contribute to disease onset and severity in patients with dyskeratosis congenita. Since this function is independent of telomerase activity, the clinical differences between patients with WRAP53β or TERT/TERC mutations might be due to accumulation of DNA damage. Indeed, mutations in WRAP53β result in a more severe form of this disease (Dokal, 2011; Ballew and Savage, 2013). Similarly, mutations in dyskerin cause a severe variant of dyskeratosis congenita with elevated numbers of γH2AX foci in response to induction of DNA double-strand breaks. The majority of these foci were not localized to telomeres, suggesting a general enhancement in DNA damage (Gu et al., 2008).

## WRAP53β takes a new route—to DNA double-strand breaks

Among the most cytotoxic DNA lesions are double-strand breaks, which are repaired by the homologous recombination (HR) or non-homologous end joining (NHEJ) pathways and involves stepwise accumulation of repair proteins at the site of damage. One of the earliest events following DNA double-strand breakage is phosphorylation of the nearby H2AX histone variant on serine-139 (to form γH2AX) catalyzed by ATM, ATR, and DNA-PK kinases (Durocher and Jackson, 2001). Next, the adaptor protein MDC1 binds γH2AX via its tandem BRCT domain and is subsequently phosphorylated by ATM. MDC1 serves as an anchor for the assembly of a variety of proteins to the site of DNA damage (Stucki et al., 2005), including RNF8, which is the first E3 ligase to be recruited to these breaks. The FHA domain of RNF8 binds to ATM-phosphorylated residues of MDC1 (four TQXF clusters). Via its RING domain, RNF8 then ubiquitylates histones H2A and H2AX at DNA damages sites, which in turn promotes accumulation of downstream factors (Huen et al., 2007; Kolas et al., 2007; Mailand et al., 2007).

**Figure 3:**
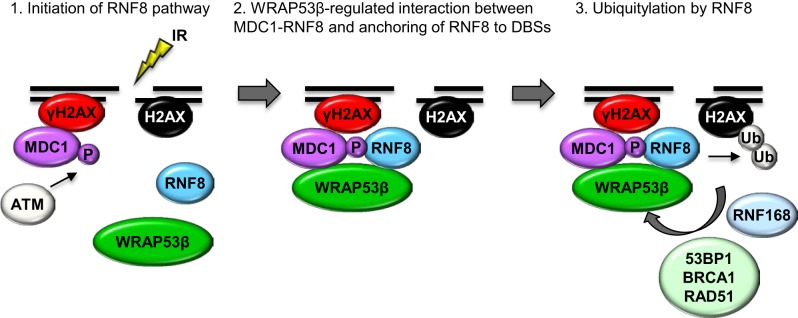
**A model of orchestration of the ubiquitin response critical for the repair of DNA double-strand breaks by WRAP53β**. Step 1: In response to IR, γH2AX and MDC1 accumulate at DNA double-strand breaks, independently of WRAP53β. ATM-mediated phosphorylation of MDC1 allows it to bind RNF8, which however, is not yet localized to the damaged site. Step 2: WRAP53β is recruited to the sites of DNA damage in an ATM-, H2AX- and MDC1-dependent manner. Simultaneous binding of MDC1 and RNF8 to WRAP53β facilitates their direct interaction as well as retention of RNF8 at the double-strand breaks. Step 3: Once assembled at double-strand breaks, RNF8 catalyzes ubiquitylation of H2AX, which enables recruitment and accumulation of 53BP1, BRCA1, and RAD51 and subsequent DNA repair.

The first indication that WRAP53β is involved in the repair of DNA double-strand breaks was its accumulation at such breaks induced by either whole cell or laser micro-irradiation. In a manner dependent on the DNA damage response protein kinase ATM, histone H2AX and MDC1, WRAP53β is rapidly recruited to DNA breaks, reaching maximal levels 1 h after damage and thereafter gradually declining as the DNA breaks are repaired. Loss of WRAP53β leads to prolonged cell cycle arrest at the G2/M stage following irradiation, as well as more spontaneous DNA breaks, which are considered to be the major cuase of genomic instability (Henriksson et al., 2014). Moreover, in cells depleted of WRAP53β recruitment of repair factors to DNA breaks is impaired and both the HR and NHEJ repair pathways are defective (Henriksson et al., 2014). Mechanistically, WRAP53β target the critical ubiquitin ligase RNF8 to DNA lesions by mediating the interaction with its upstream partner MDC1. WRAP53β binds the FHA domains of both RNF8 and MDC1 simultaneously via its WD40 domain, thereby facilitating accumulation of RNF8 and ubiquitylation at DNA double-strand breaks (Henriksson et al., 2014). Accordingly, WRAP53β is required for the assembly of downstream repair proteins such as 53BP1, BRCA1, and RAD51 at DNA breaks (Figure 3) (Henriksson et al., 2014).

## The two faces of WRAP53β in cancer

One of the effects of WRAP53β depletion observed earliest was apoptosis of cancer cells *in vitro* whereas normal cells were unaffected (Mahmoudi et al., 2011). The subsequent finding that WRAP53β is overexpressed in a variety of cancer cell lines of different origins and that such overexpression promotes carcinogenic transformation indicated that this protein possesses oncogenic properties (Mahmoudi et al., 2011). In agreement with this proposal, WRAP53β has been found to be overexpressed in primary nasopharyngeal carcinoma (Sun et al., 2014), esophageal squamous cell carcinoma (Rao et al., 2014) and rectal cancer (Zhang et al., 2012a). Moreover, knockdown of WRAP53β in cancer cells reduced the size of the tumors formed when these are grafted into mice (Sun et al., 2014).

The role of WRAP53β in telomere elongation can at least partially explain its oncogenic properties, since re-activation of telomerase is what immortalizes 90% of all human cancer (Kyo and Inoue, 2002). At the same time, knockdown of WRAP53β in cancer cells induces apoptosis within 48–72 h, whereas knockdown of telomerase enhances cell death only after several weeks in culture (Shammas et al., 2005). The observations that inactivating mutations in both alleles of WRAP53β causes dyskeratosis congenita, indicates that this protein acts as tumor suppressor, rather than an oncogene.

Indeed, with its complex roles in a number of cellular processes, WRAP53β may act as a tumor suppressor under certain conditions and as an oncogene in under others. The subcellular localization of WRAP53β may also explain some of its contradictory effects in tumor cells. For example, loss of nuclear, but not cytoplasmic WRAP53β is correlated survival and resistance to radiotherapy in patients with head and neck cancer (Garvin et al., 2015). Thus, the levels of this protein in the nucleus and cytoplasm must be considered separately in connection with patient prognosis.

Single nucleotide polymorphisms (SNPs) in the *WRAP53* gene have been linked to an increased risk for breast and ovarian cancer (Garcia-Closas et al., 2007; Schildkraut et al., 2009; Medrek et al., 2013). One of these SNPs is also associated with defective DNA repair and hematotoxicity in workers exposed to benzene. SNPs in *WRAP53* and four other genes (*BLM, WRN, RAD51*, and *TP53*), the products of which play key roles in DNA repair and maintenance of genomic integrity, especially via the HR pathway (Lan et al., 2009), have been found to predispose to bezene toxicity. At this point, it is unclear how the SNPs in *WRAP53* alter the functions of the WRAP53β protein.

In conclusion, WRAP53β dysfunction is associated with many diseases, but it is currently unknown whether accumulation of DNA damage and/or deficient DNA repair contributes to their etiology.

## WRAP53β unwraps the crosstalk between Cajal bodies, RNA processing, telomeres and the DNA damage response

DNA damaging agents, such as UV, cisplatin and IR all disrupt Cajal bodies and results in mislocalization of the marker protein coilin to microfoci and nucleoli. Moreover, coilin depletion enhances cell viability upon cisplatin treatment (Cioce et al., 2006; Boulon et al., 2010; Gilder et al., 2011). Such observations clearly reveal that the Cajal body responds to stress.

Several components of the Cajal body, including WRAP53β, have been linked to the DNA damage response. Coilin interacts with Ku70/Ku80 and inhibits NHEJ, presumably by preventing recruitment of the Ku proteins to DNA ends (Velma et al., 2010). SMN and Gemin2, members of the SMN complex, promote RAD51 assembly at DNA double-strand breaks and HR (Takaku et al., 2011). Interestingly, SMN interacts with methylated H3K79 in chromatin via its Tudor domain, a site known to target 53BP1 to DNA double-strand breaks (Huyen et al., 2004; Sabra et al., 2013). Notably, coilin also contains a Tudor domain that similar to SMN binds demethylarginine, as shown for Sm proteins (Tripsianes et al., 2011; Tapia et al., 2014).

Certain factors involved in the DNA damage response are localized in Cajal bodies. For instance in *Drosophila*, following auto-modification, PARP translocates from chromatin to Cajal bodies (Kotova et al., 2009) and WRAP53β might also shuttle between these same two compartments. Furthermore, the SUMO E3 ligase PIAS4 (also known as PIASγ), which accumulates at DNA double-strand breaks and is required for efficient RNF8-mediated ubiquitylation at sites of DNA damage, is present in the Cajal body (Sun et al., 2005; Galanty et al., 2009). A number of large-scale screens for factors involved in the DNA damage response have revealed an enrichment of proteins involved in RNA processing, although their exact involvement is not yet understood (Li and Manley, 2005; Montecucco and Biamonti, 2013).

Only future investigations can reveal whether specific components of the Cajal body contribute to DNA repair, including repair events with which WRAP53β is associated. Moreover, the impact of the RNA-related activities of WRAP53β on the DNA damage response and/or the phenotypes associated with a deficiency in this protein remains to be determined. It is also unknown whether cells of patients with spinal muscular atrophy exhibit elevated DNA damage or deficient DNA repair. In any case, since coilin depletion does not disrupt DNA repair (Henriksson et al., 2014), maintenance of the structure of Cajal body is not linked directly to this process.

Telomeres represent an important additional link between WRAP53β, Cajal bodies and the DNA damage response. DNA repair proteins are present on both functional and dysfunctional telomeres. In the case of functional telomeres, these factors promote homeostasis and prevent end-joining events. For example, DNA-PKcs appear to promote telomere capping, thereby attenuating telomere fusion (D'adda Di Fagagna et al., 2004). Moreover, Ku70/80 interacts directly with TERC to promote telomere maintenance (Ting et al., 2005). Dysfunctional, uncapped telomeres are recognized as DNA double-strand breaks by the DNA damage response, resulting in the assembly of repair factors into local telomere dysfunction-induced foci (TIFs) (Takai et al., 2003). HR and NHEJ at such sites gives rise to chromosome fusions and genomic instability, demonstrating that DNA repair at the wrong place can result in genomic instability. For example, RNF8 promotes the assembly of repair proteins at telomeres by ubiquitylating their ends, thereby facilitating chromosome fusion in cases of telomere dysfunction (Peuscher and Jacobs, 2011). Furthermore, 53BP1 increases the mobility of dysfunctional telomeres, bringing chromosomal ends into close proximity and thereby allowing NHEJ (Dimitrova et al., 2008). The findings that WRAP53β is involved in the recruitment of both RNF8/53BP1 to DNA double-strand breaks and telomerase to telomeres raises the interesting possibility that WRAP53β regulate these processes via a common mechanism.

Telomerase-deficient ALT cells are characterized by very long and heterogeneous telomeres maintained by HR. In analogy to the role of Cajal bodies in telomerase-dependent telomere elongation (Jady et al., 2006; Tomlinson et al., 2006), a specific subpopulation of telomere-associated PML bodies has been proposed to promote the recombination in ALT cells. In addition to the conventional components of the PML body, these ALT-associated PML bodies contain many proteins involved in the DNA damage response (Brault and Autexier, 2011). NBS1 is essential for their assembly: depletion of this protein leads to smaller number of ALT-associated PML bodies and telomere shortening in ALT-cells, but has no such effect on telomerase-positive cells (Wu et al., 2003; Zhong et al., 2007). Such observations indicate an intriguing link between nuclear body compartmentalization, telomere elongation and DNA damage response proteins.

## Concluding remarks and future perspectives

**Figure 4:**
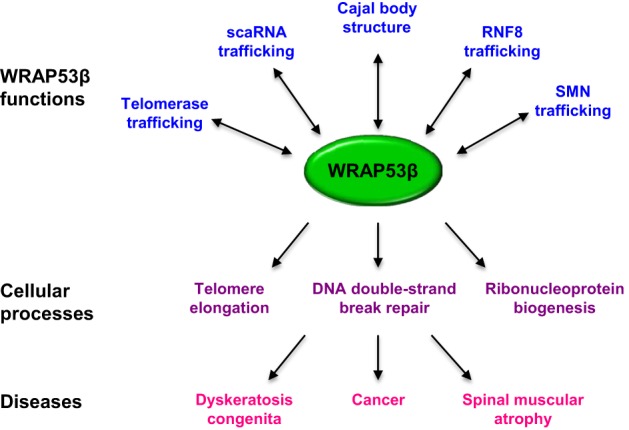
**Overview of the functions of WRAP53β and associated cellular processes and diseases**.

All of the functions described for WRAP53β involve the recruitment and proper targeting of factors to specific cellular sites (Figure 4). This protein binds the SMN complex in the cytoplasm and subsequently promotes its entry into the nucleus and localization to Cajal bodies. Patients with spinal muscular atrophy exhibit defective binding of WRAP53β to SMN, as well as reduced accumulation of SMN in Cajal bodies (Mahmoudi et al., 2010). Moreover, WRAP53β regulates telomerase localization to Cajal bodies, as well as to telomeres, and disruption of this trafficking causes dyskeratosis congenita (Venteicher et al., 2009; Zhong et al., 2011).

In addition, WRAP53β binds scaRNAs and promotes their localization to Cajal bodies (Tycowski et al., 2009). Finally, this protein binds the E3 ligase RNF8, facilitating its interaction with MDC1, which is required for its proper localization to DNA breaks and downstream repair events (Henriksson et al., 2014). Clearly, WRAP53β is as an essential scaffold protein that interacts with many types of RNA and protein, contributing both to their intracellular trafficking and interaction with other factors. However, it remains to be determined whether other Cajal body components also play a role in DNA repair, including repair associated with WRAP53β.

The discovery that that inherited mutations in *WRAP53* causes the syndrome dyskeratosis congenita, which predisposes for cancer, suggests that this is a tumor suppressor gene. However, WRAP53β also possesses oncogenic properties and can be a potential target of cancer therapy (Mahmoudi et al., 2011). Disruption of Cajal bodies is expected to decrease production of mature snRNPs, resulting in inefficient splicing, and inhibitors of the spliceosome have shown anti-tumor activities (Van Alphen et al., 2009). In addition, deficient WRAP53β-mediated trafficking of telomerase results in telomere shortening, both *in vitro* and *in vivo* (Venteicher et al., 2009). Moreover, the pathways involved in DNA repair are also targets for cancer therapy, either directly or in combination with DNA-damaging agents (Helleday et al., 2008). Further insights into the physiological roles of WRAP53β and its contribution to the development of cancer might be provided by transgenic animal models.

### Conflict of interest statement

The authors declare that the research was conducted in the absence of any commercial or financial relationships that could be construed as a potential conflict of interest.
